# A follow up report validating long term predictions of the COVID-19 epidemic in the UK using a dynamic causal model

**DOI:** 10.3389/fpubh.2024.1398297

**Published:** 2024-09-09

**Authors:** Cam Bowie, Karl Friston

**Affiliations:** ^1^Retired, Somerset, United Kingdom; ^2^Queen Square Institute of Neurology, University College London, London, United Kingdom

**Keywords:** dynamic causal model, COVID-19 mitigation measures, acute-post COVID-19, hospital admissions, mortality incidence

## Abstract

**Background:**

This paper asks whether Dynamic Causal modelling (DCM) can predict the long-term clinical impact of the COVID-19 epidemic. DCMs are designed to continually assimilate data and modify model parameters, such as transmissibility of the virus, changes in social distancing and vaccine coverage—to accommodate changes in population dynamics and virus behavior. But as a novel way to model epidemics do they produce valid predictions? We presented DCM predictions 12 months ago, which suggested an increase in viral transmission was accompanied by a reduction in pathogenicity. These changes provided plausible reasons why the model underestimated deaths, hospital admissions and acute-post COVID-19 syndrome by 20%. A further 12-month validation exercise could help to assess how useful such predictions are.

**Methods:**

we compared DCM predictions—made in October 2022—with actual outcomes over the 12-months to October 2023. The model was then used to identify changes in COVID-19 transmissibility and the sociobehavioral responses that may explain discrepancies between predictions and outcomes over this period. The model was then used to predict future trends in infections, long-COVID, hospital admissions and deaths over 12-months to October 2024, as a prelude to future tests of predictive validity.

**Findings:**

Unlike the previous predictions—which were an underestimate—the predictions made in October 2022 overestimated incidence, death and admission rates. This overestimation appears to have been caused by reduced infectivity of new variants, less movement of people and a higher persistence of immunity following natural infection and vaccination.

**Interpretation:**

despite an expressive (generative) model, with time-dependent epidemiological and sociobehavioral parameters, the model overestimated morbidity and mortality. Effectively, the model failed to accommodate the “law of declining virulence” over a timescale of years. This speaks to a fundamental issue in long-term forecasting: how to model decreases in virulence over a timescale of years? A potential answer may be available in a year when the predictions for 2024—under a model with slowly accumulating T-cell like immunity—can be assessed against actual outcomes.

## Background

Dynamic causal modelling (DCM) stands apart from most modelling in epidemiology by predicting *mitigated* outcomes and quantifying the uncertainty associated with those outcomes (1–3). This contrasts with quantitative epidemiological forecasts that do not consider the effect of prevalence on sociobehavioral responses. Usually, epidemiological projections are over few weeks—and rest upon fitting curves to the recent trajectory of various data; e.g., (4). DCM considers what is most likely to happen based upon a generative model that best explains all the data available. This mandates a model of sociobehavioral responses that mitigate viral transmission, such as social distancing, lockdown, testing and tracing, etc. In turn, this requires a detailed consideration of how various sorts of data are generated. For example, it has to model fluctuations in testing capacity and sampling bias due to people self-selecting when symptomatic. The advantage of this kind of modelling is that any data generated by the model can be used to inform the model parameters that underwrite fluctuations in latent states, such as the prevalence of infection. Latent states refer to those states of the population that cannot be estimated directly and have to be inferred from observable data.

In October 2022, the predictions carried out 12 months earlier using a Dynamic Causal model were assessed and found to underestimate the waves of new COVID-19 infections in the period October 2021 to October 2022 by 43%, deaths by 20%, tests by 24%, hospital admissions by 31% and long COVID by 21% (5). This method of modelling besides predicting health outcomes can also estimate changing characteristics of the epidemic, such as the properties of viral transmission, immunity induced by vaccine or infection, and the propensity to leave home thereby increasing the risk of catching the infection. We concluded that the underestimation of predictions could be explained by the arrival of the Omicron variants and the changes in public health policies in the UK (6–8).

This paper is a sequel to the previous paper which, besides seeking to validate the previous 12-month predictions, makes predictions to October 2023. It sets out to assess the underlying properties of the epidemic during that period from October 2022 to October 2023. It also seeks to predict what will happen in the 12 months to October 2024 assuming the current properties of the epidemic remain as they are in October 2023. We take the opportunity to provide predictions under priors based upon recent empirical estimates of latent, incubation and infectious periods. In 2024, the accuracy of predictions should speak to the usefulness of constraining parameter estimates with informative (empirical) priors of this sort.

This article can be read as a technical report, following up on previous reports, in which certain predictions were made. We anticipate a follow-up report evaluating the predictions made in this article over the forthcoming year, which will also provide an overall synthesis of long-term forecasting with dynamic causal modelling. This report provides the opportunity to compare long-term forecasts with what actually happened over timescales of years. We therefore take the opportunity to compare the predictions and actual outcomes quantitatively. Crucially, this comparison is in the latent state space of the causes of epidemiological (and behavioral) measurements. In other words, because we are using a generative or forward model of the epidemic, we can revisit the predicted fluctuations in time-dependent epidemiological and behavioral parameters *in the light of post-hoc estimates using the same model*. This effectively identifies where prior assumptions about key time-dependent parameters were not endorsed by empirical outcomes. This may be useful for future modelling initiatives along these lines.

## Methods

### Dynamic causal models

The dynamic causal model (DCM) used in this research has been continually updated with data as the epidemic has unfolded. It is designed to allow modification of model parameters, such as transmissibility of the virus, changes in social distancing, and vaccine coverage—to accommodate changes in population dynamics and virus behavior. A recent model (26th September 2023) was used to explore the effect of changing transmission of the various Omicron variants and the likely seasonal effect of the coming winter. One modification was tightening the constraints on changes in antibody immunity over time. The potential benefit of a successful Find, Test, Trace, Isolate and Support scheme was also incorporated into the model.

#### General and specific features of DCMs

The general and specific features of Dynamic Causal Models have been described in our previous publication (9). Since October 2022 our DCM COVID-19 model has been updated 20 times with the recent update on 26^th^ September 2023 (10).

### Data sources and assumptions

16 of the 24 data sources used in the model and in our previous report have been discontinued (Supplementary Table S1):

- UKHSA COVID-19 data dashboard (11)

o Deaths within 28 days of COVID-19 infection – June 2023
o Critical care bed admissions – May 2023
o Hospital occupancy of COVID-19 cases – May 2023
o COVID-19 antibody tests – October 2022

- Office of National Statistics (12)

o Deaths by age – July 2023
o Vaccinations by age – July 2023

- UK Government dashboard - Mobility – April 2022
- Google mobility Report (13)– October 2022
- IHME estimate of Incidence (confirmed and non-confirmed cases) – April 2023 (14)

The UK Government COVID-19 dashboard still provides eight key input variables such as confirmed cases, hospital admissions, certified deaths, tests and vaccine coverage (11). The Office of National Statistics (ONS) discontinued the Coronavirus (COVID-19) infection survey in March 2023 (15) which had provided the best estimates of incidence using routine antibody tests and symptom questionnaires on a regular basis to a random population sample.

The trend in the use of non-pharmaceutical interventions by the UK government is measured using the Oxford Tracker stringency index (7). The incidence of long COVID is calculated using the findings of a global meta-analysis of post-acute COVID-19 syndrome (with defined clusters of self-reported symptoms occurring 3 months after initial infection) which found the risk of long COVID following symptoms in the community is 7.9%, in hospital admissions is 27.9% and ARDS (acute respiratory distress syndrome) is 41.4% (16). The image of the proportion of variants in circulation used in Figures K-T is taken from Our World in Data (17) which uses data sourced from Gisaid (18).

For the predictions to October 2024, it is assumed that mitigation efforts such as improved ventilation in schools and workplaces will not take place, that lockdown will not be re-imposed, and that no new more virulent variants will arrive.

### Model priors

To predict outcomes over the next year, the model was run using the latest available data and prior estimates used by the DCM dashboard (19). To address the predictive validity of empirical priors we ran the model to furnish predictions with changes to the prior estimates of the model parameters, where recent research suggests appropriate values. These empirical priors were as follows: prior time constant for the latent period is 5.5 days and for the incubation period is 6.5 days in line with the results of a recent meta-analysis (20). The infectious period is given a prior time constant of 4.3 days in line with a recent paper (21), Table 1 [mean growth phase 1.6 days; mean decline phase 2.7 days]. Supplementary Table S2 provides a comparison of the priors that maximize model evidence and the new (empirical) priors.

**Table 1:** Cumulative numbers of COVID-19 cases, deaths, tests, hospital admissions and post COVID-19 Syndrome – 1st February 2020 – 1st October 2023 and 12 month projected numbers for 1st October 2023–2024 – UK.

Scenario assuming FTTIS is 25% effective	DCM 2022 projection	Actual	Data source	DCM 2023 projection
Cumulative totals from 1st February 2020 to	1st October 2023	1st October 2023		1st October 2023 to 1st October 2024
Estimated incidence	485,603,813	131,242,140	IHME - 1 Apr 2023	40,692,662
Confirmed cases by PCR and LFT	53,409,837	24,743,787	Our World in Data - 30 Sep 2023	524,351
Deaths within 28 days of a positive PCR test	330,957	229,765	Our World in Data - 30 Sep 2023	24,100
Tests (both PCR and LFD)	821,181,901	602,512,524	UK Covid-19 dashboard - 30 Sep 2023	14,080,675
Hospital admissions	1,867,580	862,553	UK Covid-19 dashboard - 30 Sep 2023	175,303
Post COVID-19 Syndrome	4,726,602	1,734,000	ONS Infection survey - 30 Mar 2023	3,139,699

For completeness, three scenarios were modelled to identify the likely effect of improving the Find Test Trace Isolate Support (FTTIS) system from a baseline of 25% effective to 40 and 60% effective.

## Findings

### Comparing projected with actual COVID-19 deaths, cases, tests, hospital admissions and incidence of long COVID

Last year’s projections overestimated incidence three-fold, confirmed cases two-fold, deaths and tests by 1.4 times, hospital admissions by 2.2 times and long COVID by 2.7 times (Table 1). The actual estimates of incidence and long COVID are only available for the first half of the year but the overestimates will still be substantial.

**Figure 1:**
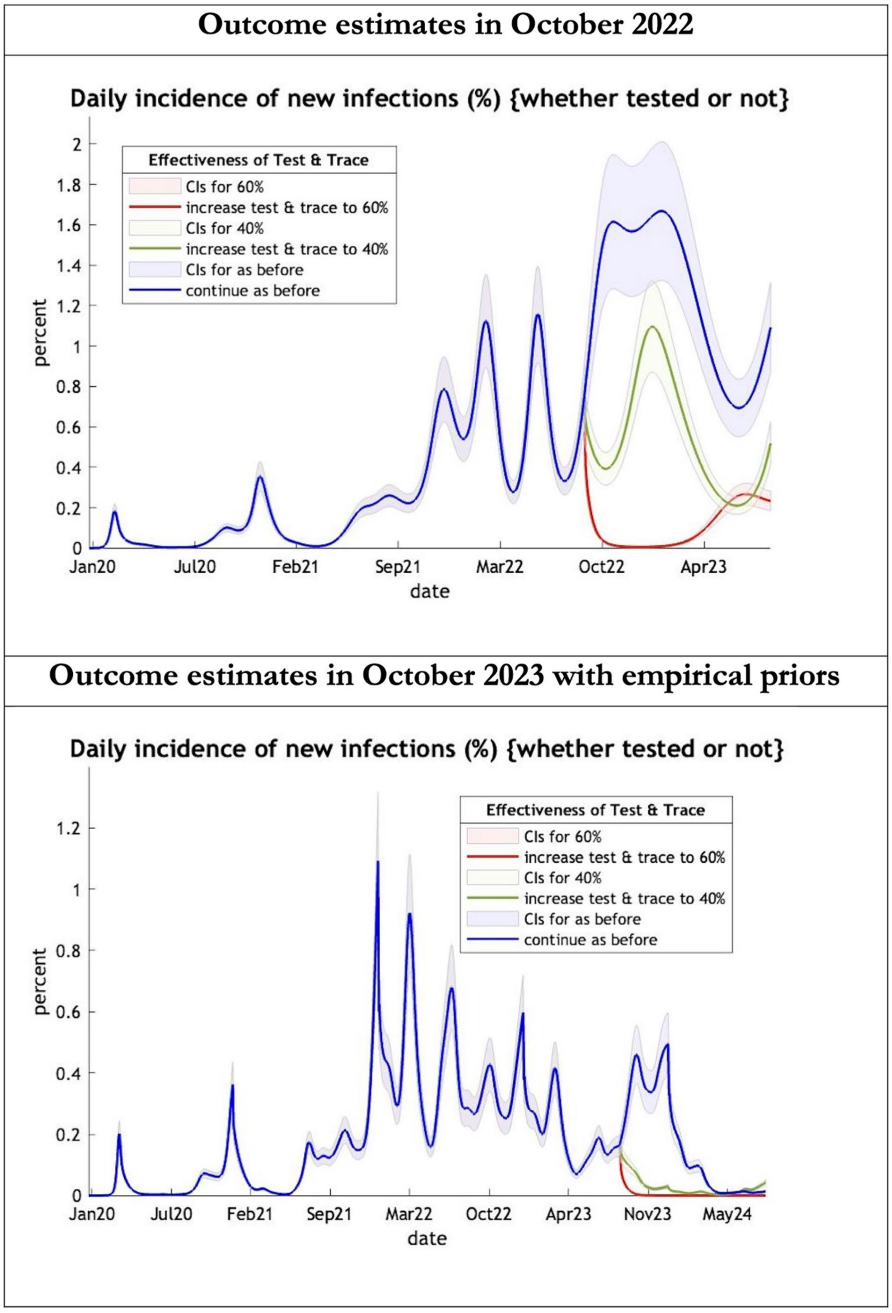
Epidemic curves of COVID-19 incidence from Jan 2020 – UK estimated by a DCM on two occasions (October 2022 and October 2023). The model can estimate incidence including cases not tested; each figure offers three projections: blue if the contact tracing system remains at 24% effective, green if it improves effectiveness to 40% and red to 60% from 1st October 2022 in the top graph and 1st October 2023 in the bottom graph: CIs, 90% credible intervals. Interpretation: The predictions with October 2022 priors are more than double the predictions using empirical priors in October 2023.

**Figure 2:**
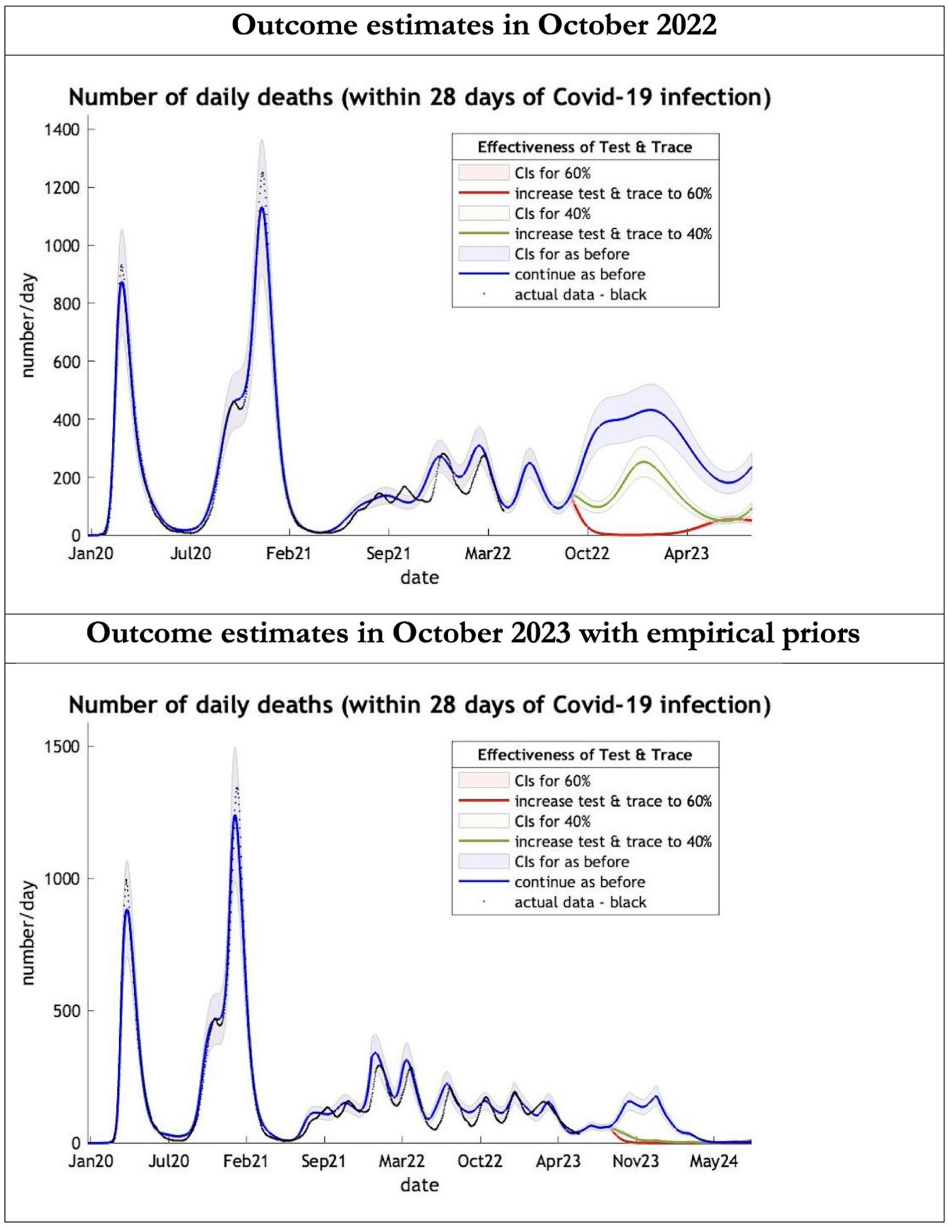
Epidemic curves of COVID-19 mortality from Jan 2020 – UK estimated by a DCM on two occasions (October 2022 and October 2023). The model can estimate projections of daily mortality certified as occurring within 28 days of a positive COVID-19 test; actual data in black is shown up till 16 June 2023—the last day of available data; each figure offers three projections: blue if the contact tracing system remains at 24% effective, green if it improves effectiveness to 40% and red to 60% from 1st October 2022 in the top graph and 1st October 2023 in the bottom graph: CIs, 90% credible intervals. Interpretation: The model is able to ape the empirical mortality series closely; the model with empirical priors offers a prediction which is half the 2022 estimates in the Oct 22 to Oct 23 period.

**Figure 3:**
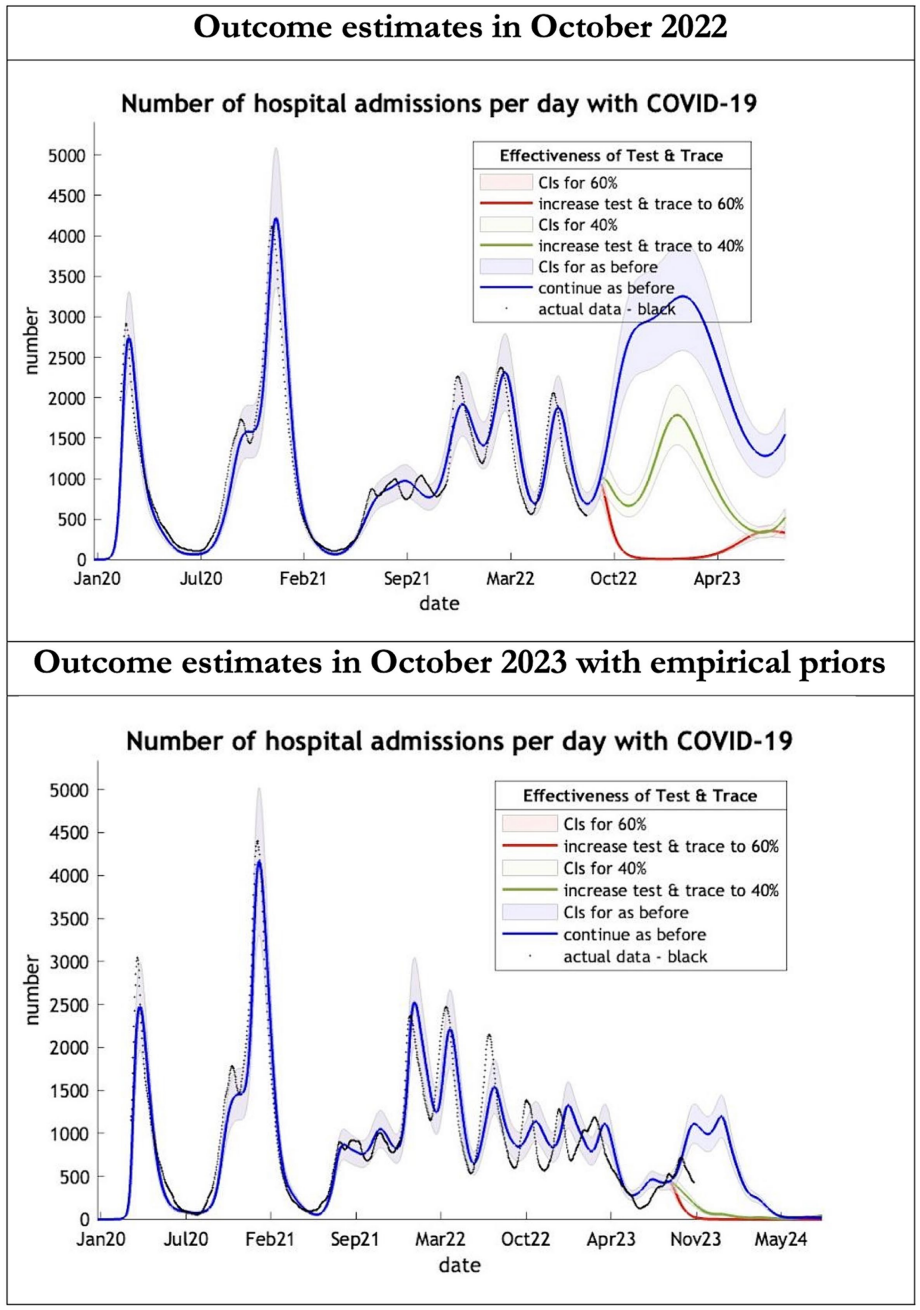
Epidemic curves of COVID-19 hospital admissions from Jan 2020 – UK estimated by a DCM on two occasions (October 2022 and October 2023). The model estimates number of hospital admissions; actual data in black; each figure offers three projections: blue if the contact tracing system remains at 24% effective, green if it improves effectiveness to 40% and red to 60% from 1st October 2022 in the top graph and 1st October 2023 in the bottom graph: CIs, 90% credible intervals. Interpretation: The 2022 estimates follow the available actual data closely until August 2022 and predicted a much larger admission rate than what occurred later. The 2023 predictions with up-to-date priors got the admission rate more or less right.

**Figure 4:**
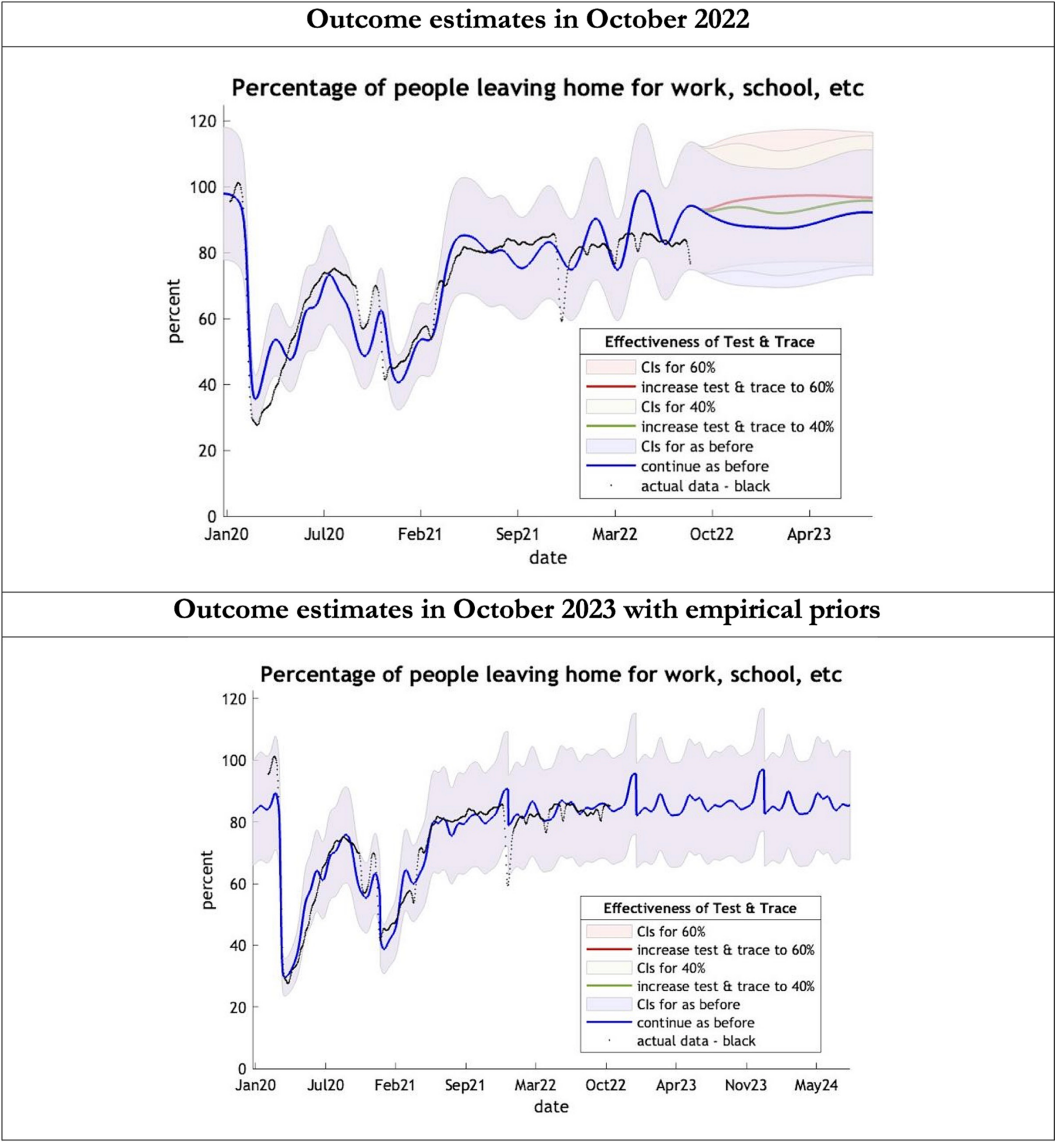
Epidemic curves of COVID-19 mobility from Jan 2020 – UK estimated by a DCM on two occasions (October 2022 and October 2023). The model estimates the number of people leaving home each day; actual data in black taken from Google Global Mobility Report; the top figure offers three projections: blue if the contact tracing system remains at 24% effective, green if it improves effectiveness to 40% and red to 60% from 1st October 2022 in the top graph and 1st October 2023 in the bottom graph: CIs, 90% credible intervals. Interpretation: The model is able to ape the actual data with exceptions in Dec 2021. The empirical 2023 priors model is able to moderate the swings in estimates seen in the model using the 2022 priors.

**Figure 5:**
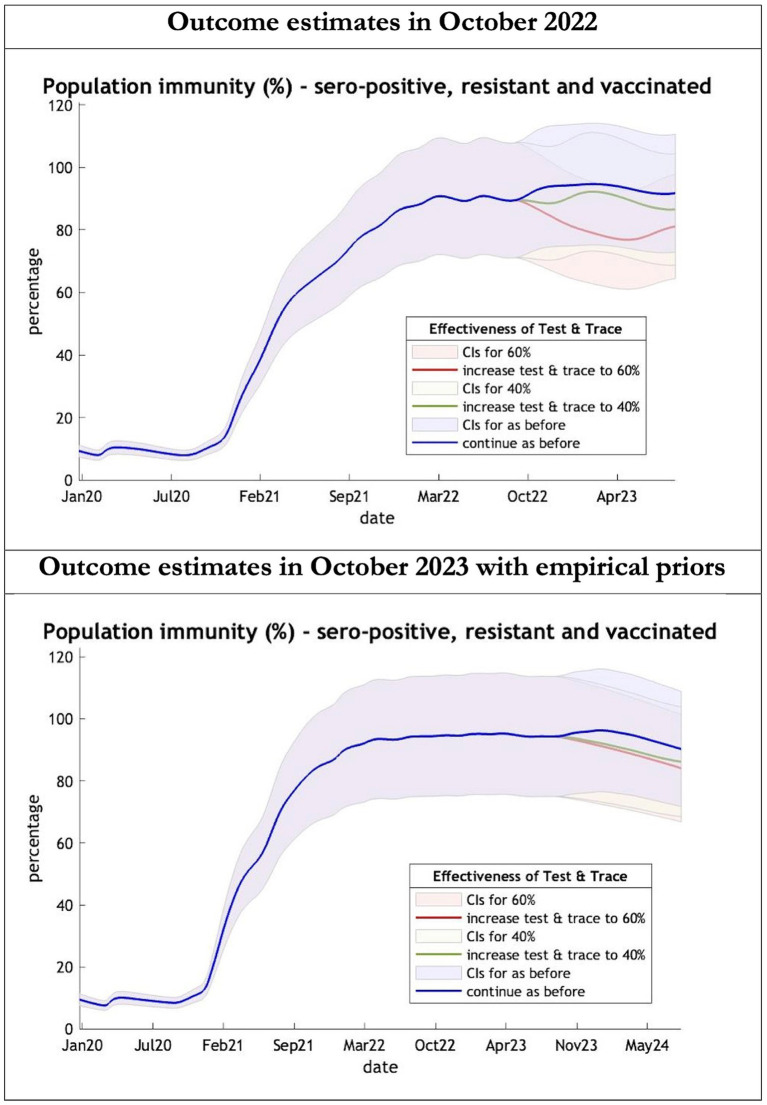
Epidemic curves of population immunity to COVID-19 from January 2020 – UK estimated by a DCM on two occasions (October 2022 and October 2023). The model estimate of population immunity to COVID-19 (% of population) including that induced by infection, natural resistance and immunization; each figure offers three projections: blue if the contact tracing system remains at 24% effective, green if it improves effectiveness to 40% and red to 60% from 1st October 2022 in the top graph and 1st October 2023 in the bottom graph: CIs, 90% credible intervals. Interpretation: Both models share similar estimates of population immunity. Neither have been able to take into account the probable declining virulence over years found in pandemics with novel viruses.

The reasons for the overestimations are found in the following two sets of graphics, which compare various outcome and parameters of model results made in October 2022 with and without knowledge of the course of the epidemic over the recent 12-month period to October 2023 (Figures 1–5). In other words, we were able to compare the time course of key epidemiological parameters estimated with and without the data covering that period (from October 2022 until October 2023). The discrepancy between these predicted and post-dictive estimates provides one account of the overestimates above.

The key overestimate was the projected large spike of infections over the winter period of 2022/2023 which did not materialize (Figure 1, top graph). Instead, we had continuous spikes of infection at lower numbers than in the previous year (Figure 1, bottom graph). The winter wave was predicted to be accompanied by large numbers of deaths and hospital admissions which did not materialize (Figures 2–3). In short, the predicted winter wave was much greater than what transpired, partly due to a projected high level of mobility (i.e., contact rates) (Figure 4) and despite a sustained level of immunity (Figure 5).

**Figure 6:**
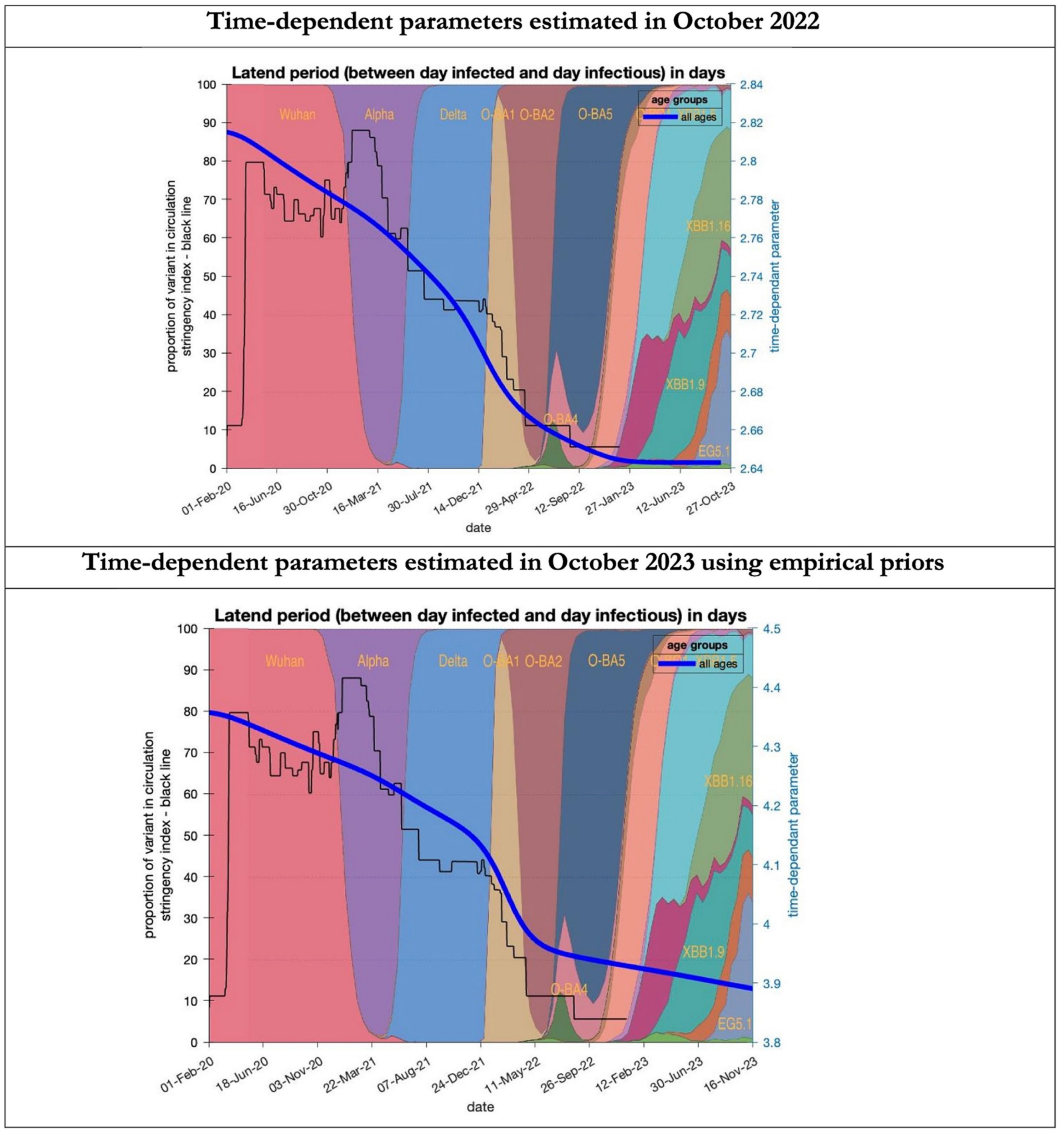
Changing estimates of latent period of COVID-19 infection in relation to the emergence of new variants and changes in response to public health policies: UK February 2020 to October 2023. Latent period (between day infected and day infectious) is measured as time constant for all age groups combined; prior in top graph of 3 days with initial model estimate of 2.8 days (infected period - Supplementary Table S2) dropping to 2.64 by October 2023; prior in the bottom graph of 5.5 days with initial model estimate of 4.36 dropping to 3.9 by November 2023; stringency index dropping from 80% in March 2020 to 5% by December 2021; proportion of variant in circulation as backdrop showing variants from the original Wuhan variant in 2020 to Omicron strains more recently (reproduced from Our World in Data). Interpretation: The variants have evolved to increase infectivity by reducing the latent period between the day infected and the day infectious. This has occurred in the both models whatever the original prior assumption used.

**Figure 7:**
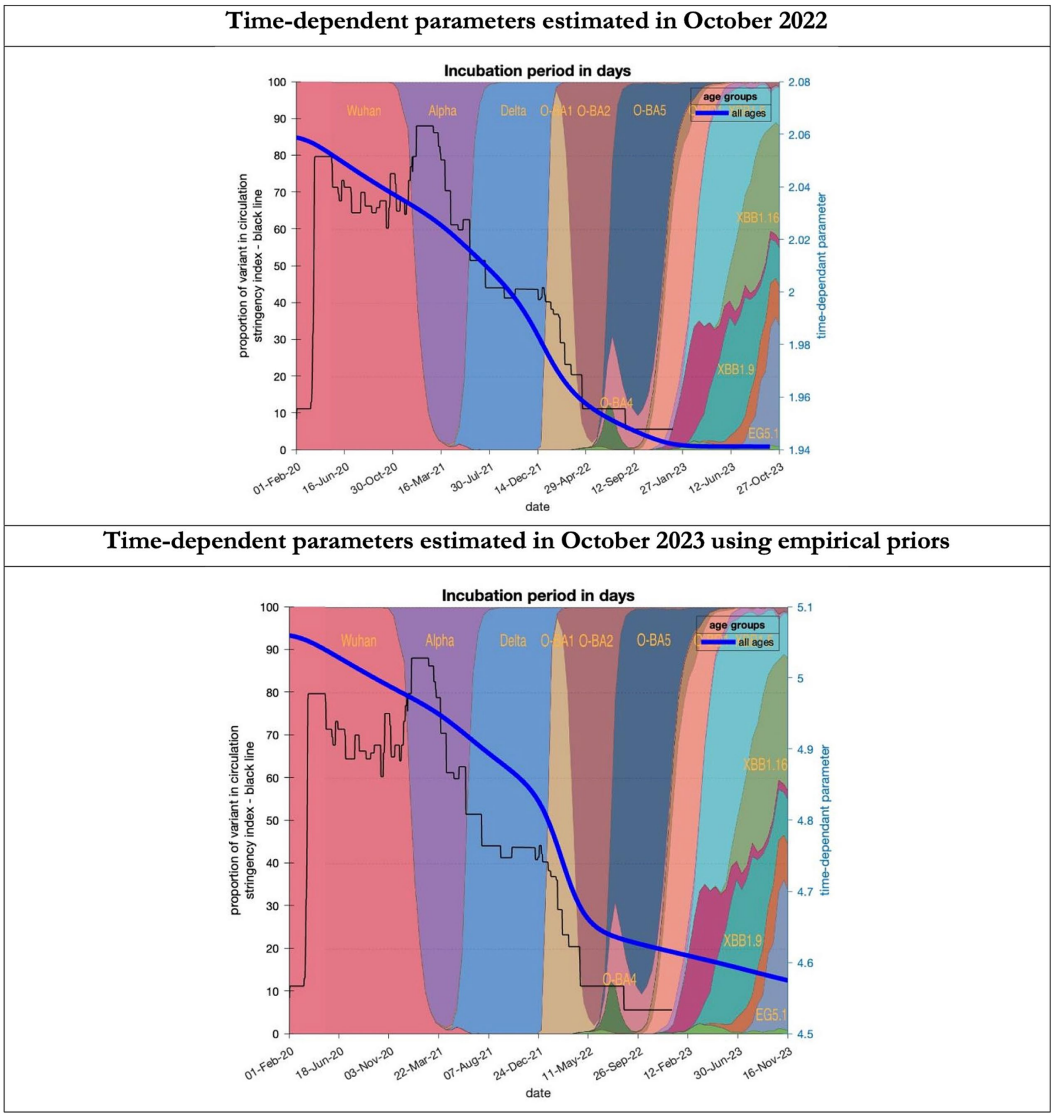
Changing estimates of incubation period of COVID-19 infection in relation to the emergence of new variants and changes in response to public health policies: UK February 2020 to October 2023. Incubation period (between day infected and start of symptoms) is measured as time constant for all age groups combined; prior in top graph of 4 days with initial model estimate of 2.06 days (asymptomatic period - Supplementary Table S2) dropping to 1.94 by October 2023; empirical prior in the bottom graph of 6.5 days with initial model estimate of 5.06 dropping to 4.6 by November 2023; stringency index dropping from 80% in March 2020 to 5% by December 2021; proportion of variant in circulation as backdrop showing variants from the original Wuhan variant in 2020 to Omicron strains more recently (reproduced from Our World in Data). Interpretation: As with the latent period the incubation period has shrunk in both models indicating the evolution of the variants which became more infectious.

**Figure 8:**
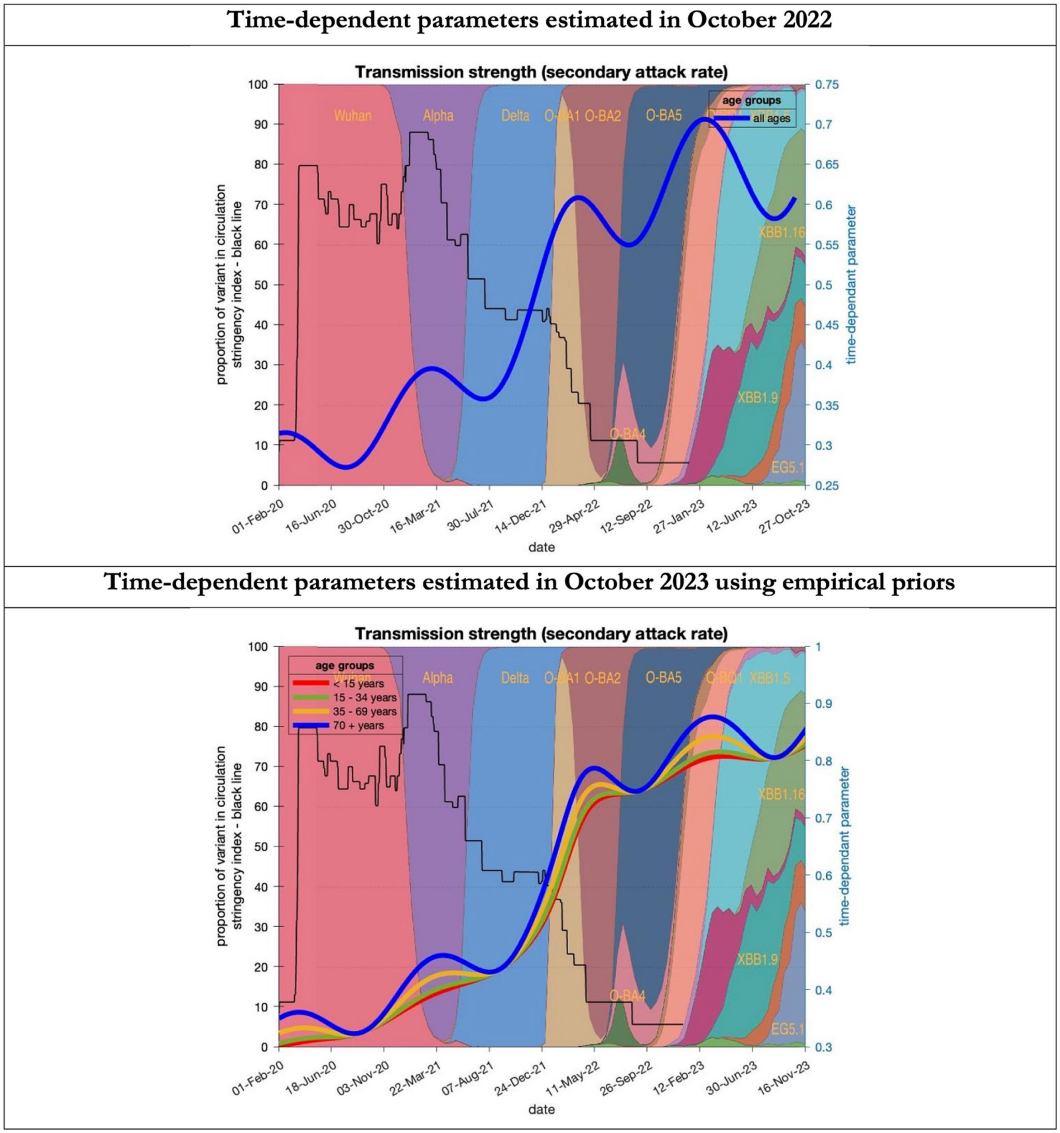
Changing estimates of transmission strength of COVID-19 infection in relation to the emergence of. Transmission strength is measured as the secondary attack rate; prior value of 0.3 (i.e, an infected person infects 1 in 3 contacts) which rises with the new variants to 0.7 (i.e. an infected person infects 70% of contacts); top graph combines all ages, bottom graph estimates transmission strength for each age group; stringency index dropping from 80% in March 2020 to 5% by December 2021; proportion of variant in circulation as backdrop showing variants from the original Wuhan variant in 2020 to Omicron strains more recently (reproduced from Our World in Data). Interpretation: Despite the different prior assumptions in the two figures the increase in transmission strength is evident in both models.

**Figure 9:**
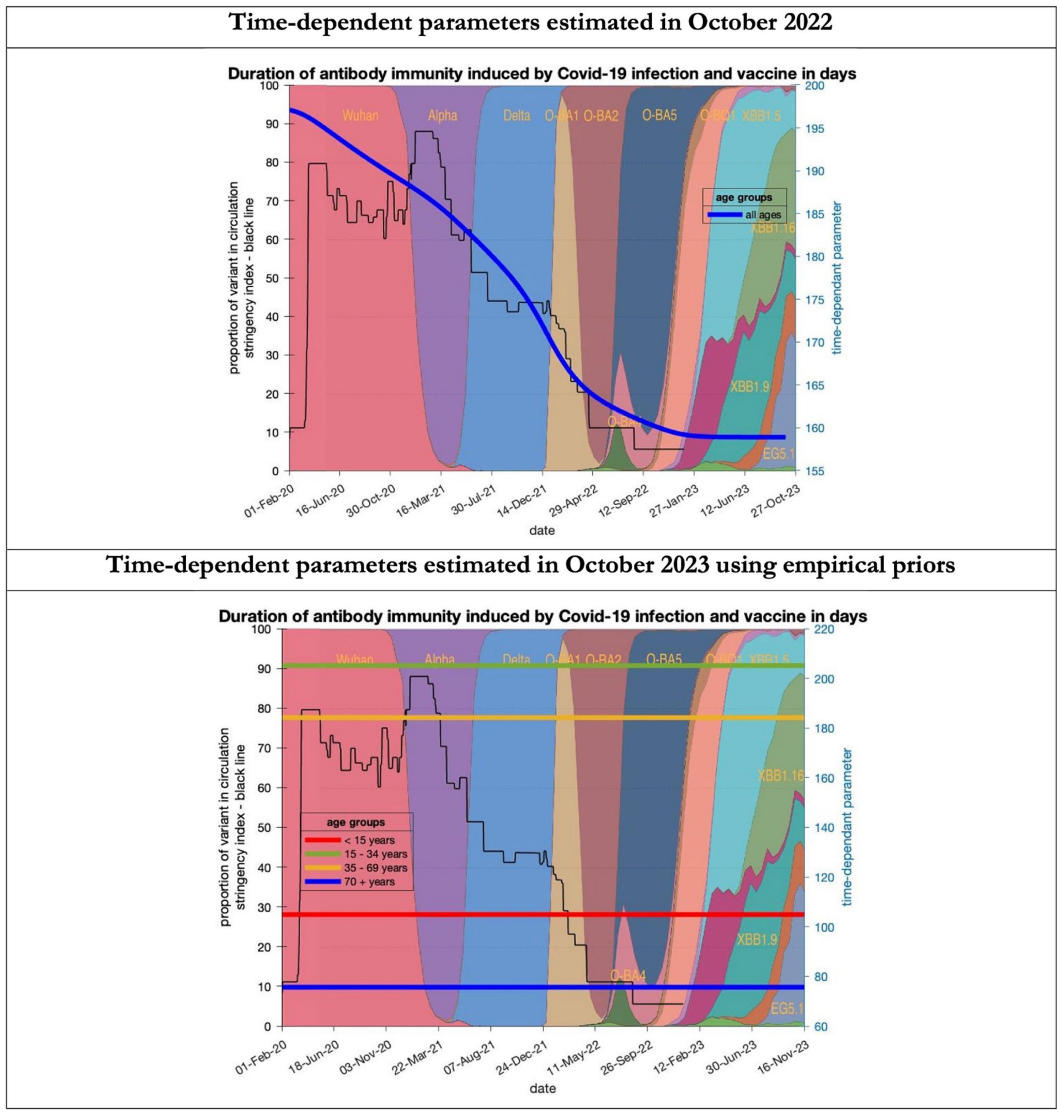
Changing estimates of duration of antibody immunity induced by COVID-19 infection and vaccine in days in relation to the emergence of new variants and changes in response to public health policies: UK February 2020 to October 2023. Duration of antibody immunity induced by COVID-19 infection and vaccine measured as time constant for all age groups combined in top graph and by age group in bottom graph; with initial model estimate of 196 days falling to 160 days by October 2023 in top graph; model estimates for each age group in bottom graph maintained at those values throughout the period; proportion of variant in circulation as backdrop showing variants from the original Wuhan variant in 2020 to Omicron strains more recently (reproduced from Our World in Data). Interpretation: The model used in 2022 assumed the possible time related change in the antibody immunity parameter whereas the 2023 model assumes no change. Further empirical data will be required to understand the changes in antibody immunity over time.

**Figure 10:**
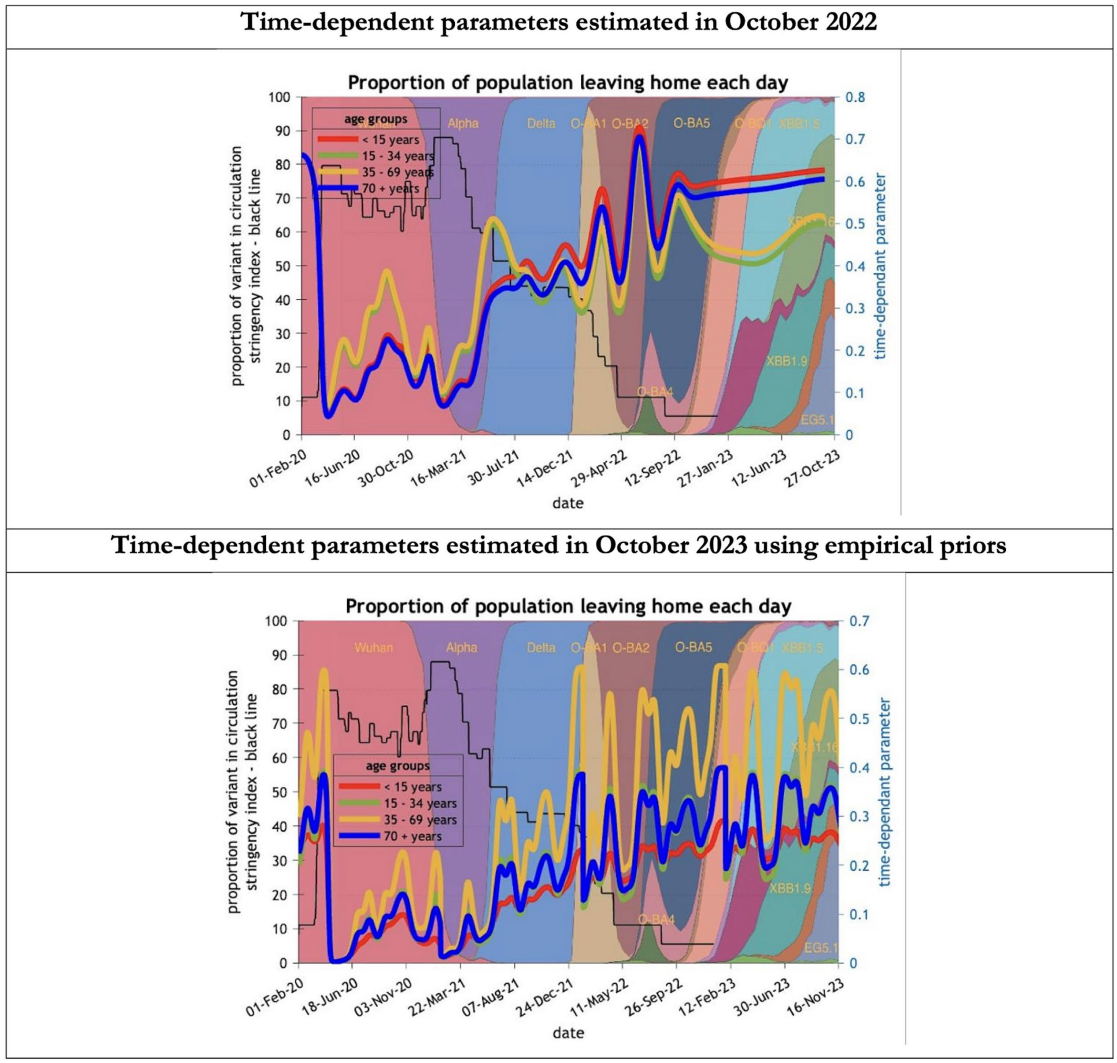
Changing estimates of the proportion of people leaving home each day in relation to the emergence of new variants and changes in response to public health policies: UK February 2020 to October 2023. The proportion of people leaving home each day for each age group; for example for those aged 70 years and above the top graph shows an estimate of 66% leaving home prior to the epidemic falling to 5% at first lockdown and rising slowly to 60% by October 2023; in the bottom graph the initial estimate for the same age group was 23% leaving home falling to 0% at the first lockdown rising to 32% by October 2023; stringency index dropping from 80% in March 2020 to 5% by December 202; proportion of variant in circulation as backdrop showing variants from the original Wuhan variant in 2020 to Omicron strains more recently (reproduced from Our World in Data). Interpretation: The 2023 predictions estimate a much less mobile population than the 2022 model. This could partly explain the overestimate of infections identified in the earlier model.

To understand the overestimates, one can look at the trajectory of the time-dependent parameters used for both predictions (Figures 6–10). The post-hoc or post-dictive estimates showed a tiny reduction but starting at a much longer starting point of 4.4 as compared to 2.8 days in the latent period (Figure 6). The incubation period, however, was longer than originally anticipated, falling not to 1.94 days but only to 4.6 days from a starting point of 5.1 as compared to 2.1 days (Figure 7). Transmission strength had increased from each infected person infecting 1 in 3 contacts to infections to infecting 80% of contacts (Figure 8). What may also be key is the change in expected antibody persistence, falling in the original from 197 to 159 days but assumed to remain constant in the late model with a posterior prior value of 105 days (Figure 9). Another key difference is the less than expected rise in the proportion of people leaving their homes, for example with only 30% of the older adult leaving home as compared to 60% in the earlier model (Figure 10). Unfortunately, the empirical data stopped at the start of the 12-months under review so we cannot be sure of the actual level of movement. By March 2023 18% of people were still wearing face masks outside and 11% in public transport (22) and 14% of adults avoided contact with vulnerable people, so it is likely that mobility increased but did not return to pre-pandemic levels.

### Future predictions

For the period October 2023 to October 2024 the model was used to predict the cumulative effect of the epidemic on case numbers, deaths, tests, hospital admissions and long COVID (Table 1 and Figures 1–3). The predictions using empirical priors suggest a wave this coming winter but with few deaths and tests but still plenty of hospital admissions and long COVID patients. Under the empirical priors COVID-19 cases will fall but still over 40 million cases and over 3 million long-COVID cases will occur in next the 12-month period. The effect of a more efficient Test and Trace system would have little influence in reducing cases using either set of priors (Figures 2, 3).

## Discussion

The overestimates of the 12-month projections to October 2023 seem to relate to better retained immunity from previous infections and vaccines at the same time as a reduction in the trend of the new variants becoming more infectious. The reason the predicted large winter wave did not occur probably relates to these factors plus a more than anticipated caution by individuals in leaving home (i.e., exposing themselves to higher transmission risk). We have no way of assessing how many infections did actually occur because the ONS infection study was stopped and estimates from other models were discontinued. Tests became infrequent and not freely available, but many particularly older adult people still observed isolation periods when thought to be infected despite pressure to ignore such practices and the removal of legal sanctions in February 2022. The year also saw antiviral therapies improve associated with a drop in case fatalities.

Finally, we have specified predictions for the upcoming year, until October 2024 based on empirical priors over the successive periods of infection. It will be interesting to see whether these empirical priors improve the model’s predictive validity.

In the next of these technical reports, we will use the current and previous reports as documentary evidence of predictions to assess the predictive accuracy of dynamic causal modelling over a forecasting timescale of weeks, months and years. We anticipate doing this by adopting the final structure of the generative model but estimating epidemiological and behavioral parameters from limited timeseries—up until a certain point in time—and assessing the posterior predictive accuracy at a series of points in the future, as the pandemic evolved. This may provide a useful reference for future pandemic modelling that leverages the unprecedented amount of data and insights generated by the COVID pandemic.

## Data Availability

The datasets presented in this study can be found in online repositories. The names of the repository/repositories and accession number(s) can be found at: https://www.fil.ion.ucl.ac.uk/spm/covid-19/. The figures in Figure 1 can be reproduced using annotated (MATLAB/Octave) code that is available as part of the free and open source academic software SPM (23). The routines are called by a demonstration script that can be invoked by DEM_COVID, DEM_COVID_X, DEM_COVID_T, DEM_COVID_I or DEM_COVID_LTLA at the MATLAB prompt. At the time of writing, these routines are available in the development version of the next SPM release. An archive of the relevant source code for each publication is available from figshare (https://figshare.com/articles/Dynamic_Causal_Modelling_of_COVID-19/12174006). The remaining results in this paper can be reproduced using modified scripts found at https://www.dropbox.com/scl/fo/zyv10xs8sn9ueuw7mhkis/h?rlkey=ewxlffkdiki89yzgjw6tz355g&dl=0. The routine data used in the manuscripts are available from the COVID-19 Data Repository by the Center for Systems Science and Engineering (CSSE) at Johns Hopkins University, Coronavirus (COVID-19) UK Historical Data by Tom White and GOV.UK Coronavirus (COVID-19) in the UK.
