# Supplementary material for: A follow up report validating long term predictions of the COVID-19 epidemic in the UK using a dynamic causal model
Source: Front Public Health. 2024 Sep 9;12:1398297. doi: 10.3389/fpubh.2024.1398297 (PMC11416950; doi:10.3389/fpubh.2024.1398297)
Supplement: Supplementary file 1 [file Table_1.DOCX]

## Supplement Table 1

The publicly available data used by the model and the date the data became no longer published.
