# Supplementary material for: A follow up report validating long term predictions of the COVID-19 epidemic in the UK using a dynamic causal model
Source: Front Public Health. 2024 Sep 9;12:1398297. doi: 10.3389/fpubh.2024.1398297 (PMC11416950; doi:10.3389/fpubh.2024.1398297)
Supplement: Supplementary file 2 [file Table_2.pdf]

number	name	description	original model parameters			recent empirical based priors		
			prior	standard deviation	posterior	prior	standard deviation	posterior
17	tin	infected period (days)	3	±0.035	2.82	5.5	±0.018	4.36
18	tcn	infectious period (days)	4	±0.045	3.63	4.3	±0.049	4.00
19	tim	loss of natural immunity (days)	128	±5	197	128	±12	104
21	tic	asymptomatic period (days)	4	±0.053	2.06	6.5	±0.018	5.06
22	tsy	symptomatic period (days)	5	±0.076	5.05	5	±0.049	10.7
23	trd	critical period (days)	16	±0.056	10.1	16	±0.135	10.4
